# Supplementary material for: Discovery of benzo[c]phenanthridine derivatives with potent activity against multidrug-resistant Mycobacterium tuberculosis
Source: Microbiol Spectr. 2024 Oct 3;12(11):e01246-24. doi: 10.1128/spectrum.01246-24 (PMC11537118; doi:10.1128/spectrum.01246-24)
Supplement: Supplemental material — Fig. S1 to S7; Tables S1 and S2. [file spectrum.01246-24-s0001.docx]

**Supplementary Information**

**Discovery of benzo[c]phenanthridine derivatives with potent activity against multidrug resistant *Mycobacterium tuberculosis***

Yi Chu Liang^1,2§^, Zhiqi Sun^2§^, Chen Lu^3,4§^, Andréanne Lupien^5,6,7,8§^, Zhongliang Xu^3,4^, Stefania Berton^2^, Peng Xu^1^, Marcel A. Behr^5,6,7,8^, Weibo Yang^3,4,9^#, Jim Sun^1,2^#

^1^Department of Microbiology and Immunology, University of British Columbia, Vancouver, Canada.

^2^Department of Biochemistry, Microbiology and Immunology, University of Ottawa, Ottawa, Canada.

^3^Chinese Academy of Sciences Key Laboratory of Receptor Research, Shanghai Institute of Materia Medica (SIMM), Chinese Academy of Sciences, Shanghai, China.

^4^University of Chinese Academy of Sciences, Beijing, China

^5^Infectious Diseases and Immunity in Global Health Program, Research Institute of the McGill University Health Centre, Montréal, Canada

^6^McGill International TB Centre, Montreal, Canada

^7^Department of Microbiology and Immunology, McGill University, Montreal, Canada

^8^Department of Medicine, McGill University Health Centre, Montreal, Canada

^9^School of Pharmaceutical Science and Technology, Hangzhou Institute for Advanced Study, University of Chinese Academy of Sciences, Hangzhou, China.

^§^Yi Chu Liang, Zhiqi Sun, Chen Lu, and Andréanne Lupien contributed equally to this article. Author order was determined in order of increasing seniority working on the project.

#Correspondence: Jim Sun, [jim.sun@ubc.ca](mailto:jim.sun@ubc.ca) and Weibo Yang, [yweibo@simm.ac.cn](mailto:yweibo@simm.ac.cn)

**Table of Contents for Supporting Information**

Figure S1-S7, Table S1-S2, and References

**Figure S1.** Structure of BPD compounds.

**Figure S2.** NMR spectra of 9-(dimethylamino)-2-methoxy-5-methylbenzo[c]phenanthridin-5-ium chloride (**BPD-6**): yellow solid, **^1^H NMR** (400 MHz, DMSO-*d_6_*): δ 9.59 (s, 1H), 8.77 (d, *J* = 9.0 Hz, 1H), 8.29 – 8.20 (m, 3H), 8.11 (d, *J* = 2.4 Hz, 1H), 7.77 (d, *J* = 2.3 Hz, 1H), 7.59 (dd, *J* = 9.3, 2.2 Hz, 1H), 7.55 (dd, *J* = 8.9, 2.3 Hz, 1H), 4.78 (s, 3H), 4.04 (s, 3H), 3.37 (s, 6H). **^13^C NMR** (126 MHz, DMSO): δ 158.02, 156.04, 151.57, 136.47, 133.51, 133.20, 131.12, 130.30, 129.67, 125.48, 124.56, 119.74, 118.74, 118.38, 115.79, 108.32, 100.88, 56.25, 50.47, 40.82. **HRMS** (ESI) calcd. for C_21_H_21_N_2_O [M ^+^]: 317.1648, found: 317.1648. Yield for two steps 52%. Total yield 2.6%.

**Figure S3.** NMR spectra of 3-(dimethylamino)-12-methyl-[1,3]dioxolo[4',5':4,5]benzo[1,2-c]phenanthridin-12-ium chloride (**BPD-9**): yellow solid, **^1^H NMR** (400 MHz, DMSO-*d_6_*): δ 9.52 (s, 1H), 8.79 (d, *J* = 9.0 Hz, 1H), 8.25 – 8.14 (m, 3H), 7.79 – 7.71 (m, 2H), 7.57 (dd, *J* = 9.3, 2.2 Hz, 1H), 6.33 (s, 2H), 4.68 (s, 3H), 3.36 (s, 6H). **^13^C NMR** (126 MHz, DMSO): δ 156.11, 151.75, 149.06, 148.51, 136.71, 133.96, 133.57, 133.29, 129.21, 123.08, 120.41, 119.68, 118.16, 115.44, 105.88, 104.56, 103.06, 100.57, 50.49, 40.89. **HRMS** (ESI) calcd. for C_21_H_19_N_2_O_2_ [M ^+^]: 331.1441, found: 331.144. Yield for two steps 46%. Total yield 1.2%.

**Figure S4.** BALB/c mice (*n*=4) were intra-peritoneally (*i.p.*) injected with BPD-9 every other day for 14 days, and the mouse body weight was recorded before each injection. The cohort injected with the highest dose of BPD-9 (10 mg/kg) was euthanized at day 6 due to a body weight loss >15%.

**A**

**B**

**C**

**D**

**Figure S5.** Absorbance and emission spectra scan of BPD-6 and BPD-9. Compounds were serially diluted (2.5 - 20 μM) in PBS buffer and the absorbance (A, B) or the emission (C, D) scan of BPD-6 and BPD-9 were measured. For the emission scan, the excitation wavelength was fixed at 420 nm.

**Figure S6.** Relative viability of Mtb-*lux* as indicated by relative luminescence units (RLU). Mtb-*lux* was treated with 105 μM gentamicin or buffer alone for 24 h in PBS buffer.

**Figure S7.** BPD-6 and BPD-9 are active against virulent and clinical Mtb strains. Dose-dependent activity of sanguinarine (SG), BPD-6, BPD-9, rifampicin (RIF), and isoniazid (INH) against the Mtb strains (Table 3): H37Rv (A), HN878 (B), and Erdman (C), clinical isolate #50 (D), #105 (E), #116 (F), #151 (G), and #217 (H) was determined using the REMA assay. Mtb viability is normalized to maximal bacterial growth in the absence of compounds as 100%. Data represent the mean ± SD of 4 replicates.

| **Strain name** | **Modifications / Resistance** | **Source** |
| --- | --- | --- |
| Mtb mc^2^6206 | H37Rv derivative with Δ*panCD*Δ*leuCD* | (1) |
| Mtb-RFP | Mtb mc^2^6206 expressing tdTomato | (2) |
| Mtb-*lux* | Mtb mc^2^6206 transformed with pMV306hsp+LuxG13 | (3) |
| *M. kansasii* Hauduroy | n/a | ATCC 12478 |
| *M. bovis* BCG (strain Institute Pasteur) | n/a | ATCC 27291 |
| *M. smegmatis* mc^2^155 | n/a | ATCC 700084 |
| Mtb H37Rv | n/a | ATCC 27294 |
| Mtb Erdman | n/a | ATCC 35801 |
| Mtb HN878 | n/a | (4) |
| Mtb MDR isolate #50 | INH^R^ | McGill International TB Centre, Montreal, Canada |
| Mtb MDR isolate #105 | INH^R^, PZA^R^, STR^R^, Other | McGill International TB Centre, Montreal, Canada |
| Mtb MDR isolate #116 | INH^R^, RIF^R^, STR^R^ | McGill International TB Centre, Montreal, Canada |
| Mtb MDR isolate #151 | INH^R^, PZA^R^, RIF^R^, STR^R^, Other | McGill International TB Centre, Montreal, Canada |
| Mtb MDR isolate #217 | INH^R^, PZA^R^, RIF^R^, ETO^R^ | McGill International TB Centre, Montreal, Canada |
| Mtb H37Rv R1_BPD-9 | *rv2678c*: C728T (p.His243His);  *rv2933*: A2685AC (p.Glu898fs);  *rv2962c*: C700T (p.Thr234Ile);  *rv3066*: G44T (p.Arg15Leu) | This study |
| Mtb H37Rv R2_BPD-9 | *rv1960*: C152T (p.Arg51Gly);  *rv3066*: G469A (p.Gly157Ser) | This study |
| Mtb H37Rv R3_BPD-9 | *rv2933*: A2685AC (p.Glu898fs);  *rv2962c*: C700T (p.Thr234Ile);  *rv3066*: C345G (p.Tyr115*) | This study |
| Mtb H37Rv R4_BPD-9 | *rv1960*: C152G (p.Arg51Gly);  *rv3066*: T197C (p.Leu66Pro) | This study |
| Mtb H37Rv pKM444 | H37Rv containing the pKM444 plasmid (KAN^R^) for gene deletion | This study |
| Mtb H37Rv Δ*rv3066_5* | Mtb H37Rv pKM444 transformed with the targeting oligonucleotide (Table S2) and the payload plasmid pKM496 (ZEO^R^) | This study |
| Mtb H37Rv Δ*rv3066_9* | Mtb H37Rv pKM444 transformed with the targeting oligonuclotide (Table S2) and the payload plasmid pKM496 (ZEO^R^) | This study |
| Mtb H37Rv Δ*mmr*-*rv3066_2* | Mtb H37Rv pKM444 transformed with the targeting oligonucleotide (Table S2) and the payload plasmid pKM496 (ZEO^R^) | This study |
| Mtb H37Rv Δ*mmr*-*rv3066_8* | Mtb H37Rv pKM444 transformed with the targeting oligonucleotide (Table S2) and the payload plasmid pKM496 (ZEO^R^) | This study |
| *E. coli* NEB Stable | n/a | New England Biolabs, Ipswich, MA |
| *P. aeruginosa* PA01 | n/a | Gift from Dr. Thien-Fah Mah, University of Ottawa |
| *P. aeruginosa* PA14 | n/a | Gift from Dr. Thien-Fah Mah, University of Ottawa |
| *S. enterica* Typhimurium SL1344 | n/a | Gift from Dr. Subash Sad, University of Ottawa |
| *L. monocytogenes* 10403s | n/a | Gift from Dr. Subash Sad, University of Ottawa |

**Supplementary Table S1.** Bacterial strains used in this study.

| **Name** | **Sequence (5’-3’)** | **Reference** |
| --- | --- | --- |
| kanR-F | tactcctgatgatgcatggttact | This study |
| kanR-R | taatttcccctcgtcaaaaataag | This study |
| rv3066  (recombineering) | cgtggtcacgttgaacctggcgggtgcccattgaccgcaggctccgaccgccgtccacgcggtttgtctggtcaaccaccgcggtctcagtggtgtacggtacaaacctctgcccatgcgggacgaacccccgactgacaccgcagcggctcccaccaccggtgcggc | This study |
| mmR-rv3066 (recombineering) | acatgtacaaatgtacacaaaggaggggtcttgatctacctatacctcttgtgcgcgatcggtttgtctggtcaaccaccgcggtctcagtggtgtacggtacaaacctctgcccatgcgggacgaacccccgactgacaccgcagcggctcccaccaccggtgcggc | This study |
| ∆rv3066-F | gtgggctgtctagtgggtta | This study |
| ∆rv3066-R | accatcttgccgttgtagac | This study |
| ∆mmr-Rv3066-F | tcaccatgacacgacgttac | This study |
| ∆mmr-Rv3066-R | agaccatcttgccgttgtag | This study |
| qPCR_mmR-F | atacctcttgtgcgcgatct | This study |
| qPCR_mmR-R | agcgatgccataacccacta | This study |

**Supplementary Table S2.** List of oligonucleotides used in this study.

**Supplementary References**

1. Sampson SL, Dascher CC, Sambandamurthy VK, Russell RG, Jacobs WR, Bloom BR, Hondalus MK. 2004. Protection Elicited by a Double Leucine and Pantothenate Auxotroph of Mycobacterium tuberculosis in Guinea Pigs. Infection and Immunity 72:3031-3037.

2. Afriyie-Asante A, Dabla A, Dagenais A, Berton S, Smyth R, Sun J. 2021. Mycobacterium tuberculosis Exploits Focal Adhesion Kinase to Induce Necrotic Cell Death and Inhibit Reactive Oxygen Species Production. Frontiers in Immunology 12.

3. Berton S, Chen L, Liang YC, Xu Z, Afriyie-Asante A, Rajabalee N, Yang W, Sun J. 2022. A selective PPM1A inhibitor activates autophagy to restrict the survival of Mycobacterium tuberculosis. Cell Chemical Biology 29:1126-1139.e12.

4. Domenech P, Rog A, Moolji J-u-d, Radomski N, Fallow A, Leon-Solis L, Bowes J, Behr MA, Reed MB. 2014. Origins of a 350-Kilobase Genomic Duplication in Mycobacterium tuberculosis and Its Impact on Virulence. Infection and Immunity 82:2902-2912.
